# Supplementary material for: Computational tools for drawing, building and displaying carbohydrates: a visual guide
Source: Beilstein J Org Chem. 2020 Oct 2;16:2448–68. doi: 10.3762/bjoc.16.199 (PMC7537382; doi:10.3762/bjoc.16.199)
Supplement: File 1 — Features of glycan sketchers, builders and viewers. [file Beilstein_J_Org_Chem-16-2448-s001.pdf]

BEILSTEIN JOURNAL OF ORGANIC CHEMISTRY

Supporting Information

for

Computational tools for drawing, building and displaying carbohydrates: a visual guide

Kanhaya Lal, Rafael Bermeo and Serge Perez

Beilstein J. Org. Chem. **2020,** *16,* 2448–2468. doi:10.3762/bjoc.16.199

Features of glycan sketchers, builders and viewers

License and Terms: This is a supporting information file under the terms of the Creative Commons Attribution License (https://creativecommons.org/licenses/by/4.0). Please note that the reuse, redistribution and reproduction in particular requires that the authors and source are credited.
The license is subject to the *Beilstein Journal of Organic Chemistry* terms and conditions: (https://www.beilstein-journals.org/bjoc)

Table S1. Schematic summary of important features of glycan sketchers, builders and viewers.

S.No.	Software Tool	2D sketchers and builders																					
													3D Builders							3D (representation) viewers			
		Sugar Sketcher	LiGraph	Glyco Glyph	Glycan Builder2 SNFG	Sugarbind Glycan Builder	Draw RingS	Draw Glycan SNFG	Glycano	Glyco Editor	Glyco.me Sugar Builder	KegDraw	Polys Glycan Builder	Sweet	CHARMM Gui	Glycam Carbo Builder	do Glycans	Rosetta Carbohydrate	Carb Builder	3D-SNFG	PyMOL	Sweet UnityMol	LiteMol
1	Available online	✓	✓	✓		✓	✓	✓	✓	✓	✓		✓	✓	✓	✓					✓		✓
2	Download for local installation	✓		✓	✓			✓	✓			✓					✓	✓	✓	✓	✓	✓	✓
3	Instructions		✓	✓	✓	✓	✓	✓				✓	✓	✓	✓	✓		✓	✓	✓	✓	✓	✓
4	Self-explanatory interface	✓		✓	✓	✓	✓	✓	✓	✓	✓	✓	✓		✓	✓						✓	
5	Image output (file type)	.SVG	.SVG	.SVG	.PNG, .SVG	.PNG .SVG .BMP …	.PNG (save as)	.JPG (save as)	.PNG .SVG	(screen shot)	.PNG .SVG	.PNG	.SVG		(screen shot)	.GIF (save as)				.BMP	.PNG	.PNG	.PNG
6	SNFG colours	CMYK	CMYK	RGB	RGB		CMYK	RGB		RGB	RGB		CMYK		CMYK					CMYK	RGB		RGB
7	Oxford linkage geometry	✓			✓	✓	✓	✓	✓		✓		✓										
8	Functionalization	✓		✓	✓	✓		✓	✓	✓		✓			✓	✓	✓	✓			✓		
9	Repeating units [ ]	✓			✓	✓	✓	✓	✓	✓		✓	✓								✓		
10	Linking as glyco-conjugates				✓		✓	✓				✓					✓	✓	✓	✓	✓		✓
11	Text input	✓	✓	✓	✓	✓	✓	✓	✓				✓	✓		✓	✓	✓	✓				✓
12	Option to modify by coding	✓	✓	✓			✓	✓					✓	✓		✓				✓	✓		
13	3D-model visualization												✓	✓	✓	✓				✓	✓		✓
14	3D model output												✓	✓	✓	✓	✓	✓	✓	✓	✓	✓	
15	Text/String output (any format)	✓	✓	✓	✓	✓	✓			✓		✓	✓	✓	✓	✓			✓	✓			
16	Glycan library	✓		✓	✓	✓	✓			✓				✓							✓		
17	No. of templates (monosaccharides)	70+	15+	80+	70+	50+	70+	60+	60+	40+	10+	10+	100+	40+	20+	30+				40+			40+
